# Evaluating COVID-19 decision-making in a humanitarian setting: The case study of Somalia

**DOI:** 10.1371/journal.pgph.0000192

**Published:** 2022-03-16

**Authors:** Abdihamid Warsame, Mohamed Fuje, Francesco Checchi, Karl Blanchet, Jennifer Palmer

**Affiliations:** 1 Faculty of Epidemiology and Population Health, The London School of Hygiene & Tropical Medicine, London, United Kingdom; 2 Office of the Chief Medical Officer, Federal Ministry of Health and Social Services, Mogadishu, Somalia; 3 Faculty of Medicine, Geneva Centre of Humanitarian Studies, University of Geneva, Geneva, Switzerland; 4 Faculty of Public Health and Policy, The London School of Hygiene & Tropical Medicine, London, United Kingdom; Universitas Gadjah Mada, INDONESIA

## Abstract

The global COVID-19 pandemic is unprecedented in its scope and impact. While a great deal of research has been directed towards the response in high-income countries, relatively little is known about the way in which decision-makers in low-income and crisis-affected countries have contended with the epidemic. Through use of an a priori decision framework, we aimed to evaluate the process of policy and operational decision-making in relation to the COVID-19 response in Somalia, a chronically fragile country, focusing particularly on the use of information and the role of transparency. We undertook a desk review, observed a number of key decision-making fora and conducted a series of key informant and focus group discussions with a range of decision-makers including state authority, civil society, humanitarian and development actors. We found that nearly all actors struggled to make sense of the scale of the epidemic and form an appropriate response. Decisions made during the early months had a large impact on the course of the epidemic response. Decision-makers relied heavily on international norms and were constrained by a number of factors within the political environment including resource limitations, political contestation and low population adherence to response measures. Important aspects of the response suffered from a transparency deficit and would have benefitted from more inclusive decision-making. Development of decision support tools appropriate for crisis-affected settings that explicitly deal with individual and environmental decision factors could lead to more effective and timely epidemic response.

## Introduction

While epidemics of infectious disease continue to pose a considerable threat to populations in low-income and crisis settings, evaluations of these epidemic responses are limited and heterogeneous [1]. Populations in need of humanitarian assistance or living in low income settings continue to grow [2] and the ongoing COVID-19 pandemic has further exacerbated the harmful impacts of these crises [3].

Somalia is a fragile state with a particularly weak health system [4]. With a population of just over 15 million, the nation has faced a protracted crisis due to ongoing insecurity, recurrent droughts and natural disasters, and frequent epidemics. The COVID-19 epidemic has strained the health system in Somalia and left wide ranging economic [5], social and political [6] impacts. As of November 15 2021, the country had reported 22,837 confirmed cases and 1313 deaths [7] while recent research points to a significantly greater excess death toll [8]. While calls are beginning to surface around the world for inquiries and evaluations [9–11] around all facets of COVID-19 response, none so far has been conducted in Somalia or, to our knowledge, other low-income or crisis settings. Among the few previous studies of epidemic decision-making in low income countries, most have been conducted in high-countries [12] or after the conclusion of an epidemic [13].

Decision-making in the context of an ongoing epidemic is particularly challenging [14,15]. Previous research has highlighted the important role that prioritization of political and economic considerations play how this can lead to delayed or ineffective response [16–18]. However, these findings can be strengthened by further study to confirm their applicability to a wider array of contexts. In particular, in order to better understand and ultimately improve epidemic decision-making, it would be useful to identify a generic model for in low-income or crisis-effected contexts, taking into account the actors, process and the contextual factors.

## Aim and objectives

The overall aim of the study was to evaluate the process of policy and operational decision-making in relation to the COVID-19 response in Somalia.

The specific objectives were to: (i) Describe who made the decisions, and how technical, security and political considerations as well as individual preferences influenced decision-making within this setting; (ii) Explore how these decisions affected the timeliness and performance of the response; (iii) Propose a conceptual model to describe policy and operational decision-making in epidemics in low-income and crisis-affected settings.

## Methods

### Ethics approval and consent to participate

Ethical approval for this study was obtained from the Federal Ministry of Health and Social Services of Somalia (Ref: MOH&HS/DGO/0994/Aug/2020) and the ethics review committee of the London School of Hygiene & Tropical Medicine (Ref: 22778 /RR/21778). Informed written consent was taken from all key informants and focus group participants.

### Study design

This study built on work conducted for the London School of Hygiene and Tropical Medicine’s COVID-19 help desk in April-November 2020 during which we advised humanitarian and government actors in Somalia and participated in a number of high-level meetings where the response was discussed. We then undertook a qualitative study from November 2020 to March 2021 utilizing a combination of direct observation, remote and in person interviews, and focus group discussion, supplemented by a desk review of response documentation. Participants were purposively sampled to reflect a broad range of experiences and views of different levels of the epidemic response in Somalia (Table 1).

**Table 1:** Data collection by method and type of participant or setting.

Primary Data collection	Type of participant or setting	Number
Key informant interviews	Civil Society Members	3
	Donor staff	2
	Federal Government staff	6
	International Committee of the Red Cross (ICRC)/ International Federation of Red Cross and Red Crescent (IFRC)	2
	Independent Experts	3
	Nongovernmental Organization staff	5
	Regional Government staff	2
	United Nations staff	8
Focus Group Discussion	Donor staff	3
Meeting Observation	Health Cluster Coordination meeting	2
	Chief Medical Officer Advisory meeting	4
	Risk Communication and Community Engagement meeting	1
	National COVID-19 coordination meeting	1

### Data collection

#### Individual key informant and focus group discussions interviews

31 key informant semi-structured interviews and one focus group discussion with 3 donors were conducted in English or Somali by the first author in both Somalia and Kenya. Data collection was capped when tracking of key themes identified during data collection suggested saturation had been reached. Written informed consent was acquired prior to any interviews. Interviews were recorded for transcription and analysis purposes. Each interview took approximately 30–60 minutes and respondents were given the option of complete or partial anonymity in which they agreed for their role or organization to be published. About half of respondents either chose to stay anonymous or requested that some portion of the interview be off the record in order to preserve important working relations. Non-English interviews were recorded, and transcribed in Somali. They were then translated into English with samples excerpts back-translated by the second author to ensure accuracy of translation.

#### Document review and observations

A review of response documents such as surveillance records, meeting minutes, operational plans and organograms was conducted to provide context, identify key decision points and potential key informants as well as to construct a response timeline. Documents were identified through grey literature search (S1 Search Strategy) as well as solicited from key informants. Additionally, news sources in English and Somali were reviewed to gain an understanding of the social, economic and political context in which the epidemic response is occurring and provided some topics for interviews.

#### Direct observation

Additionally, we observed decision-making within, eight national response meetings between April–November 2020. These included meetings of the humanitarian health cluster mechanism, the national coordination meetings of government and civil society and technical meetings to advise the chief medical officer (Table 1). Additional key coordination mechanisms including the UN Taskforce and Somalia Donor Group were identified but we were unable to attend meetings.

### Decision-making framework

**Fig 1:**
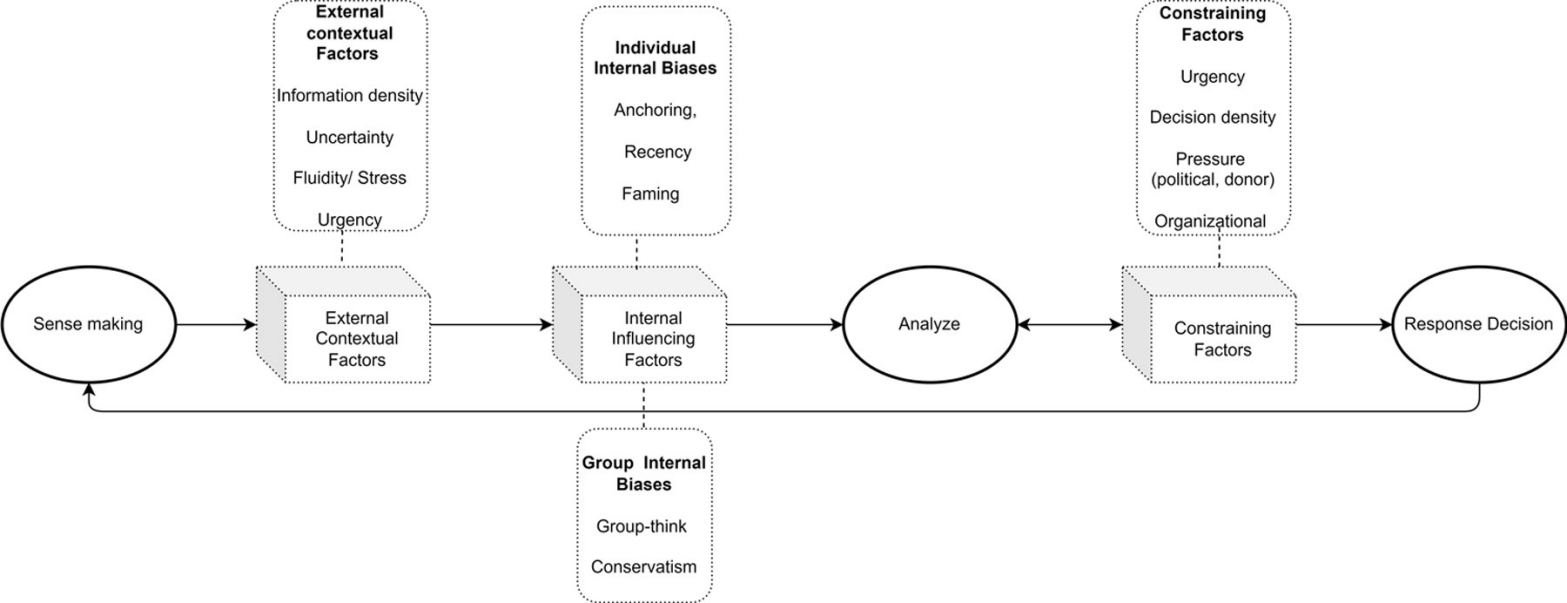
Draft decision-making framework for epidemic response.

We previously reviewed existing policy and operational frameworks for responses to public health emergencies [19] and methods used in epidemic evaluations in low-income and crisis-affected countries [1], culminating in a new proposed Adaptive Epidemic Response Framework (AERF) [19]. We approached this study by combining the AERF with the Cynefan Framework for decision-making [20] into an a priori decision-making framework (DMF) for epidemic response, whose suitability for describing decision-making in epidemics we used this study to evaluate (Fig 1).

The framework posits that epidemic decision-makers first make sense of the scenario (through gathering and assessing incoming information), then analyse (integrate and categorize new and previous information according to available disease control decision-making strategies) and finally make a decision. However, each of these steps are filtered through and constrained by several internal and external factors [21] with both constraining factors and new opportunities for sense-making influencing analysis iteratively until a decision is finally taken.

### Data analysis

Interview and focus group data were analysed using a deductive thematic approach structured by the DMF components and using Nvivo software. This approach helped explore how closely the reality of decision-making in the COVID-19 response in Somalia aligns with the DMF as well how the DMF could be adapted to reflect this reality. The analysis considered a number of potentially influential contextual factors which may have impacted on the COVID-19 response. These included the political relationships among and between state authorities, international actors (UN, INGO or donors), local actors and grassroots initiatives in the context of concurrent and recent crises (drought, food insecurity, economic crises, cholera outbreaks) and the impending 2020 contested election.

## Results

### Context, timeline and key actors

**Fig 2:**
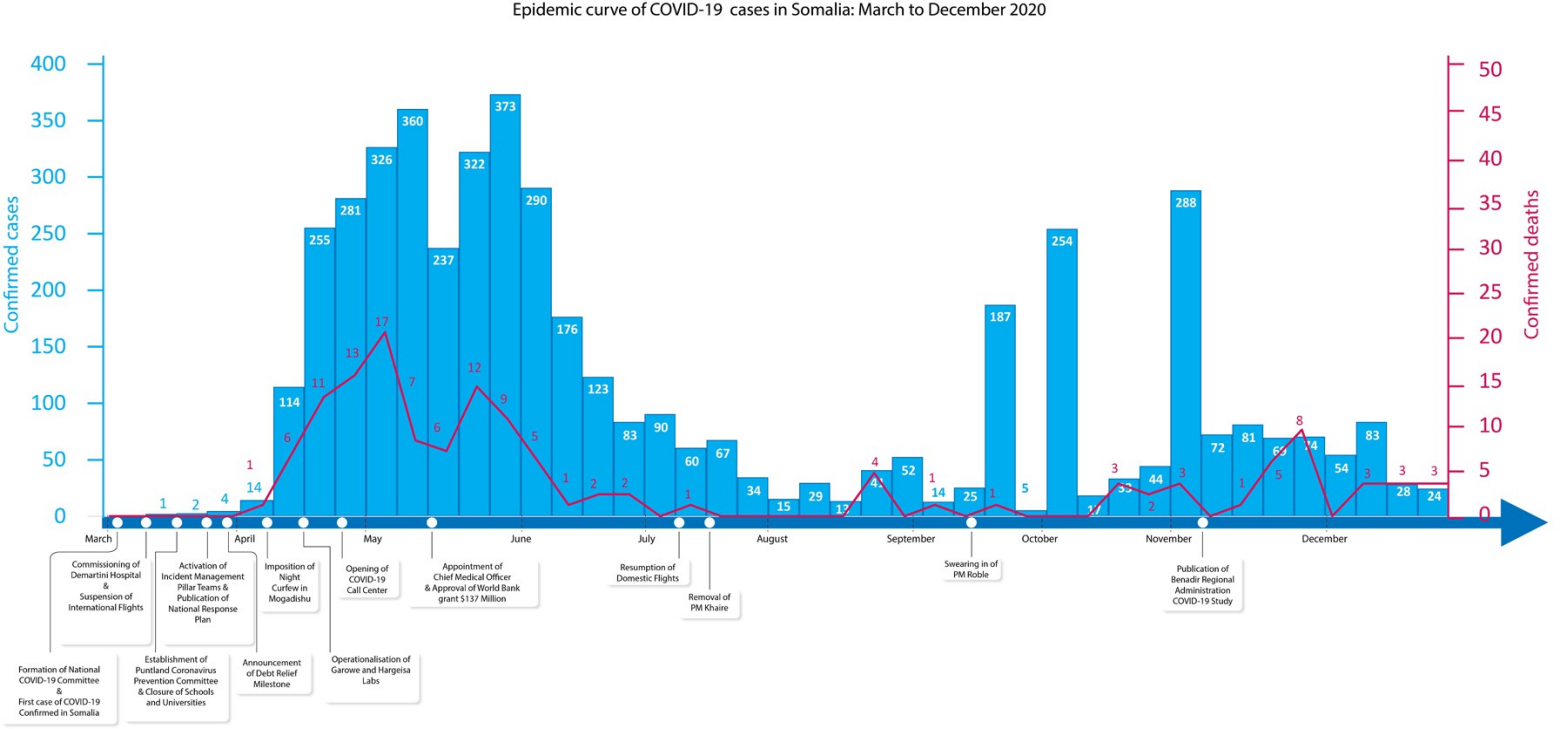
Timeline of key events and corresponding official weekly epidemic curve Mar-Dec 2020. Case data from Ministry of Health Federal Republic of Somalia and WHO Somalia.

The first COVID-19 case was confirmed in Somalia on March 15, 2020 (Fig 2) in the midst of increased climactic shocks, chronic insecurity [22] and a once in a generation locust infestation [23]. Against this backdrop, the epidemic response coordination in Somalia was undertaken through existing as well as newly formed mechanisms.

As Somalia had a long history of public health emergencies, a large number of humanitarian and health actors were already in place supporting the public health system. These included seven specialized UN agencies, a multitude of local and international NGOs, federal and regional ministries of health, and several bilateral and multilateral donors.

Among the existing coordination mechanisms, clusters (groupings of humanitarian organisations working within the same thematic area) as well as the Humanitarian Donor Group (comprised of representatives of donor agencies) remained active and continued their role. The health cluster is an open forum for coordinating among all national and international organizations implementing health programming within Somalia while the Humanitarian Donor Group is a closed forum limited to bilateral donors.

UN agencies under the overall leadership of the WHO and pillar specific leadership of various UN agencies also activated a new incident management pillar system of coordination, previously utilized in other outbreaks [24] with the ministry of Health operating a similar incident management structure [25].

The epidemic occurred at a critical time when the leadership of the country was keen to be seen as having turned a corner towards political stability and economic development. The federal government had cleared debt arrears to the International Development Association (IDA) and was being considered for debt relief by the International Monetary Fund (IMF) under the heavily indebted countries mechanism [26]. If successful, this process was set to relieve the country of substantial debt, opening the door for substantial sums of development funding and signalling the international community’s confidence in the governance and direction of the nation [27]. In this context, the Prime Minister’s office was keen on centralizing high-profile projects under its direct management to increase legitimacy and protect against mismanagement. The epidemic provided an opportunity to realize both objectives.

In response to international reports of the pandemic, the Federal Government prepared a national contingency [28] plan and formed a national COVID-19 committee in the first week of March modelled on the COBRA emergency coordination mechanism in the UK. The structure of this committee was due in part to the influence of British-Somali diaspora in key advisory positions. This committee, largely seen by a majority of informants to be inclusive and effective, was comprised of several ministries including the Ministry of Health, and Ministry for Relief and Disasters, civil society, representatives of the business community and infectious disease specialists [28]. Similar committees were set up at the regional level.

Respondents mentioned that at the outset of the crisis, the national COVID-19 committee chaired by the Prime Minister took decisive action through a number of key measures including closure of airspace and education facilities, the designation of treatment facilities and the formation of a national COVID-19 call centre. However, there were some oversights for example, interstate vehicle travel continued largely unabated. Overall, these actions cemented the leadership of the committee in the early months of the epidemic with international actors perceived by some informants as playing a delayed supporting role.

The Prime Minister was generally praised by a number of informants for being responsive and playing a direct and active role in many aspects of the response. The leadership role played by WHO was likewise praised by informants in setting up and guiding the response through its leadership in the UN task force as well as its support to government and implementing agencies.

Wide scale testing was a significant challenge with the limited number of tests being sent to neighbouring Kenya for analysis [29]. In April 2020 as testing capacity was expanded and an increasing number of cases were confirmed, the national committee attempted to impose a lockdown and curfew in the capital Mogadishu in an attempt to contain the epidemic [30]. However, these attempts by the government at imposing restrictions and securitizing the response met with considerable population pushback [31]. During this time, the Prime Minister appointed for the first time a chief medical officer to provide technical advice to the committee. Shortly after, a corruption scandal came to light in the Federal Ministry of Health in which several senior civil servants were implicated in embezzling COVID-19 funds [32]. The scandal threatened to derail the much anticipated external debt cancellation by the IMF and World Bank and led the Prime Minister to take an even more direct role in the COVID-19 response for example by bringing oversight of COVID-19 funds under his office. In July domestic flights were resumed in response to a decline in cases and heavy lobbying by business groups. By this time, there was a general perception that the worst-case scenario had not materialized and that a gradual reopening as observed in high-income countries at this time was warranted. National policymaker’s attention then turned towards political contestation ahead of the national elections culminating in the removal of the Prime Minister on the 25^th^ of July. Activities of the national committee and involvement of the PM’s office lost momentum in the run-up to national elections on 25 July in which PM Khaire was removed from office. Management of the high-profile, COVID-19 call centre, for example, was handed over to the Ministry of Health. A new Prime Minister was not sworn in until the 23^rd^ of September creating a vacuum in the national COVID-19 response coordination in which no major response initiatives were launched. During the last quarter of 2020, some locally driven research began to be published on the impact of COVID-19. Despite this increase in research evidence, COVID-19 continued to fall off the policy agenda as electoral politics grew increasingly acrimonious. In February 2021, a second, more aggressive wave of infections began to emerge possibly due to the introduction of new variants.

### Sense making: Understanding the epidemic and its impact

Respondents struggled to come to grips with the epidemic due to a number of external contextual factors as well as internal drivers of individual and group decision-makers.

Previous emergencies were largely contained within the country and did not constrain the ability of international partners to source external support. However due to the global nature of the pandemic, resources for COVID-19 were not as readily available in Somalia.

*“This was the first incidence of an outbreak or this kind of situation that the global situation affected the in-country situation*.*”–*UN Official 2.

Respondents noted that the uncertainty around how to respond at a global level shaped sense-making within Somalia across several dimensions, particularly in terms of peoples’ anticipation of the scale of the epidemic.

*“We struggled for quite some time with a better assessment of how deep is this epidemic actually going in Somalia*.*”*–Donor Official 1.

There was also uncertainty on how to formulate an appropriate response in this rapidly changing information landscape.

*“It seemed like there was a bit of a crisis in terms of [identifying] how to respond to something like this that was evolving*. *The information out there kept changing*”–NGO Official 3. *“One of the difficult things early on was this flood of [new] guidance materials and international guidance*.*”–*UN Official 2.

Uncertainty was further compounded by a sense of unpreparedness which respondents attributed to a lack of appropriate pandemic planning, response structures and sufficient resourcing to deal with this crisis which was predicted to lead to a high death toll [33].

The unprecedented nature of this epidemic generated panic both in the general population and in responders. The Somali public exhibited ambivalence [34] with a large proportion of the population considering COVID-19 as non-existent or irrelevant to Somalia whilst others feared becoming infected [35,36]. This resulted in a situation in which people were simultaneously not practising preventative measures but also not using routine health services for fear of infection.

“*The service utilization from the community was not that much at the beginning of the outbreak because people were afraid*. *People were not coming to heath facilities and people were staying at home*. *“-* UN Official 5

Response organizations not only felt unequal to the magnitude of the task but staff members felt particularly vulnerable as they felt they did not have adequate information, training or equipment to deliver services while protecting themselves. There was also widespread fear among responders about how the epidemic could impact the ability of the already fragile health system to protect the public.

*“One of the things that I must acknowledge*, *that there was a lot of panic*. *There was a lot of panic amongst various agencies amongst various individuals*. *We knew that this is a fragile state*, *we knew the health system is not maybe ready*.*” -*UN Official 3

Given the widespread panic at the outset, responders noted feeling overwhelmed by the urgency to respond.

*“There was urgency in the sense that I had to get everything done before another wave came along*, *or before things escalated*. *It was almost like there was something that’s going to happen and you don’t know what’s going to happen*, *but you just got to act before it happens*. *That’s what it felt like*, *at least*.*”* -Independent Health Expert 2

Respondents cited three factors that helped them find their footing in making sense of and responding to the epidemic: relying on their organizational mandate, taking guidance from international norms and falling back on their individual experiences of epidemics in Somalia and abroad. Several respondents mentioned their organizational mandate as being their primary driver to respond to the COVID-19 epidemic. However, other organizations cited the lack of capacity in the Somali context as driving their decision to respond while under normal circumstances epidemics may not have been within their mandate. As described by a representative from the Red Cross,

“*Even though our mandate is of war and conflict and so forth*, *it became evident*, *especially in Somalia and in many other contexts and other delegations*, *that there wasn’t really a response from other organizations or they didn’t have the capacity to become as responsive as we could be*.*” -*Red Cross Official 1

The longstanding aid relationship between Somalia and several high-income countries played a significant role in framing the response to COVID-19. This relationship reinforced expectations that, as had been the case with previous emergencies, best practise and support in responding to COVID-19 would come to Somalia from abroad. This reflex-like adoption of these best practises is a potential example of group-think bias as some respondents believed local alternatives did not exist. There was consensus, particularly among respondents in government on the necessity of such an approach as they believed local data and expertise were not sufficient to the task.

*I can say most of the times*, *what was happening outside the world was influencing some of the decisions the committee and the government had to make because this was a new disease*, *it was a new virus*. *We had to learn from other countries where the virus appeared before us*.–Government Official 3.

Lastly, because of the novelty of this epidemic, many respondents relied heavily on their own personal experiences to understand the situation.

*“We were always dealing with outbreaks*: *outbreaks of meningitis*, *measles*, *outbreaks of cholera*, *they were all there*.*”—*Government Official 5.

However, drawing uncritically on such experience may lead to recency bias as respondents overestimate the relevance of recent experience to COVID-19 and therefore utilize inappropriate response measures. For example, the existence of response tools such as vaccinations and well-established treatment protocols for previous epidemics might potentially incline responders to underestimate the challenge of COVID-19 despite the absence of such tools [37].

### Analysis: Exploring options

#### Constraining factors

Respondents mentioned the already difficult humanitarian access landscape of Somalia being further compounded by COVID-19. They mentioned difficulties for responders to access locations outside of the major urban centres both in terms of receiving information as well as providing necessary response measures. Several areas were labelled ‘silent districts’ because there was a near total COVID-19 information blackout. Respondents also stated that the repatriation of key international response staff impeded their response. As one humanitarian worker supporting a remote response from Nairobi reflected:

“*I think our partners*, *as well as ourselves*, *were not able to go as often to the field*, *to Mogadishu and to the field outside Mogadishu*, *as already in normal times it’s very difficult for security reasons and logistic reasons*. *Now with the COVID pandemic*, *in addition*, *it was even more difficult to actually get around and to move around*, *and to be in contact with the beneficiaries”*. *-*Donor Official 2.

Respondents also mentioned lack of transparency around information particularly around the official epidemiological figures. This was mainly in relation to instances of discordance between regional and federal statistics as well as periods of information blackout. Additionally, some particularly impactful decisions such as national lockdowns were said to be undertaken in a non-transparent way.

*“The Ministry of Health just randomly shut down their information giving, and then the call centre was just shut down for no reason. I guess there’s almost cliff-hangers, so many cliff-hangers that are so unnecessary”–Government Official 2*.

Such unaccountable actions were seen by some respondents as undermining the rhetoric of strengthening state legitimacy that had initially guided the response at the onset of the pandemic.

Financial constraints were a critical constraining factors in designing an appropriate response. Donors, in particular, felt the humanitarian funding arena was more uncertain compared to previous emergencies and they had little leeway to make new allocations. This was both because of the long-standing problem of donor fatigue due to the chronic nature of crises in Somalia as well as the globalized nature of the COVID-19 emergency where Somalia programmes had to compete with programmes not only in low-income countries but high-income ones as well.

Furthermore, they mentioned how the population’s coping mechanisms had been overstretched from previous emergencies as well as government’s inability to soften economic burden of lockdowns as underlying opposition or non-adherence to curfews and lockdowns. Implementing agencies as well as government respondents mentioned their overwhelming reliance on international partners. This was especially troubling given that less than 2% of the federal budget was allocated for health services [38]. Respondents felt their latitude to act was severely curtailed as a result of this resource limitation.

“*Because every single country in the world was impacted including donor countries*. *It wasn’t a case of*, *‘This was all happening in low-income countries and somehow OECD countries were doing fine*.*’ The response was required in the UK as much as it was in Mogadishu*,… *This whole question of could we apply for additional resources*. *It came at a time when the economy of everyone was being impacted*. *Money was actually getting tighter*. *We actually had to reduce our budget”*. *-*Donor Official 3.

Respondents were ambivalent about whether and how the political environment in which the epidemic occurred constrained response options. Early on in the epidemic, many respondents felt that the pandemic had the effect of fostering a cohesive response and minimising political jockeying among political leaders in Somalia. As elections loomed and political actors were drawn into a lengthy period of post-election contestation, however, national political tensions tended to heighten tensions over resources, diluting focus and resources away from the response.

Discord was not solely limited to political contestation but was also recognized as key constraint in coordinated decision-making amongst responders. Primary amongst them was disagreement between government health authorities and health partners. These were mainly to do with government actors asserting their authority over health partners on issues such as the channelling of external funds directly through health authorities, controlling information channels or changing programme activity areas. In one instance an NGO informant related the discord with the government arising from the prioritization of political considerations.

“*[the government] focus too much on equality*, *but they don’t look very much on the equity [based on need]*. *They should instead let us put more resources where the pandemic is so prevalent*, *where it is high*.”–NGO Official 5.

However other respondents rationalized this approach as necessary to the stability of the state.

“*The government trying to accommodate as many people as the government can*, *comes down to the aspect of the state building trajectory in Somalia—*-Civil Society Expert 2.

There was also discord within government between federal and state authorities and among different ministries largely over resources or areas of responsibilities. Some respondents disagreed with the structuring of the response and felt that existing institutions were side-lined. The pandemic further exacerbated the existing ambiguity in mandate between the Ministry of Health and Ministry of Humanitarian Affairs and Disaster Management with introduction of new and independent response bodies such as the National COVID-19 call centre.

“*Why we are out of the blue creating new institutions when we already have two institutions whose job it is to tackle the pandemic*?*”*.*—*Civil Society Informant 1

Respondents characterised successful decision-making as being one in which disagreement in decision-making were addressed collectively at the correct level rather than implemented unilaterally. Examples given of successful collective decision making relied on two main approaches: consensus building at the lower levels and compromise through negotiations at the higher levels. At the lower or technical decision-making level, respondents mentioned that issues were thoroughly debated and consensus was sought so as to present a unified position to more senior decision-makers.

Some respondents however mentioned that decision-makers in more senior forums sometimes debated issues for which they were not technically proficient. In one meeting we observed, the national committee debated revising the lockdown strategy in response to widespread public resistance. Some non-health members of the committee lobbied for shifting all focus towards equipping and running treatment facilities as they perceived outreach activities such as prevention messaging to be ineffective. Health members of the committee supported prioritising resources towards robust community-based social-distancing and shielding measures, as well as case detection and surveillance, rather than more expensive curative approaches. Perceiving their influence to be diluted by the opinions of non-health committee members, health members responded by calling upon external technical expertise to present on the potential impacts of various response approaches through mathematical modelling. The models presented several potentially catastrophic scenarios if different response modalities were adopted including one in which only case management was undertaken. The presentation of these scenarios was acknowledged by senior committee members as lending credence to the health members’ position to continue utilizing a multipronged approach rather than focusing on a single response activity. The committee dismissed the suggestion of relying solely on case management as too risky and consensus reached on the necessity of a multipronged approach including community outreach.

On the other hand, compromise was often used for tackling the more sensitive issues. A pertinent example is the disagreement between government and religious authorities over mosque closures. This issue arose early in the response when the role of mosques as important avenues of transmission, was first debated in the national committee. The issue was raised by the Minister of Religious Affairs in the context of safeguarding the accessibility of mosques and was debated by other committee members. The chief medical officer and other health officials presented evidence that persuaded the committee of the risk that mosques posed.

*“Even the Grand Mosques in Mekkah and Madinah are closed because it is recognized risk*, *so we must do the same*.*”* Government Official 5.

Once consensus was reached on this point, the debate then proceeded to the best mitigation strategy; whether it was enforcing social distancing and limiting attendance or whether a complete closure was warranted. The committee was split on this issue, with some calling for the latter while the members of the religious community argued that this was premature given that markets and schools and other public venues remained open. A compromise was reached in which the religious community successfully argued that mosques were an essential service and should only be closed as a last resort and only when a full lockdown of other venues was in place. This compromise was illustrative of the influence wielded by religious constituents, the degree to which the government was keen not to alienate them as well as the government’s limited capacity to unilaterally enforce closure in the face of opposition. In the end, while schools were successfully closed [35], a brief attempt at a general curfew was ineffective [31] and the closure of mosques was never implemented.

While examples such as these demonstrate the deliberative nature of some high level meetings, this was not the norm in many decision-making fora. Meetings observed tended to be structured around information sharing rather than deliberation with the chair wielding large influence on the degree to which deliberations were permitted.

#### Decision-making criteria

Respondents disclosed several criteria that guided their COVID-19 response decision-making including equity, accountability, windows of opportunity, as well as state-building aims.

Respondents mentioned that the epidemic brought to the forefront the pre-existing neglect of health systems in the country. Respondents also stated that the pandemic had unequal impact on different socioeconomic segments of the population with those at the lowest socioeconomic level bearing the greatest burden.

“*Basically*, *what we realized along the way was that COVID was a very privileged disease”*. *-*Government Official 2.

This reality eventually became clear to many responders, who addressed it by utilizing an equity-based approach to allocating resources and efforts to combat the pandemic.

*“I think in terms of location by region*, *we started from the [geographic] area which is affected most”*–NGO Official 2.

Some respondents revealed the role that wider economic factors had in their decision-making for example with regards to imposing travel restrictions.

*“There is pressure from other sectors especially trade*, *commerce and they’re saying that closure of the airport cannot continue”–*Independent health expert 1

Respondents identified a general deficit in accountability in the response. They cited in particular a lack of transparency in the allocation of and utilization of response resources. This was cited as a problem at all levels including within organizations, between government bodies as well as with regards to allocation from global to national levels within individual agencies.

*“A lot of money to be distributed but absolute opacity as to [how]…And we kept asking right*, *"How much is Somali getting? How much has come to Somalia? What are you buying with that money?” Donor Official 3*

Much of the discussion around accountability centred on the importance of financial accountability to donors as well as the lack of consideration given to accountability to affected populations. Some actors felt that the lack of transparency in an already complex political environment was a critical factor in poor decision-making, suggesting that a decision support tool that could explicitly address decision criteria might be helpful in future epidemics.

In weighing the options presented by various decisions, respondents often considered whether a decision presented a new opportunity to further an existing aim. Respondents stated that the epidemic focused attention on the health arena and presented new opportunities to raise funds to strengthen Somalia’s chronically weak health system.

“*One of the advice that we gave was we have to use COVID as an entry point to build a resilient health system for not only COVID but also for future pandemics and epidemics*.*”*–Independent Health Expert 1.

Some respondents mentioned that some actors were more interested in highlighting their response activities rather than on their impact.

“*From the way it seemed like*, *it seemed as if it was a point-scoring opportunity to say*, *“Look*, *we are doing this great work*. *Look at us*. *Look at us*.*"—*Independent health expert 3.

Many respondents stated that the epidemic presented an opportunity to underscore legitimacy and credibility on the part of some decision-makers. In particular, this was seen as a key motivation for government authorities.

“*The Office of the Prime Minister coming in and taking charge of the response efforts was sending a picture to the Somali public and to the Somali people that the government is sparing no efforts to respond*.*”*–Civil Society Expert 2.

Many respondents also considered state building as a crucial criterion in undertaking a response with all respondents stating their activities were done with the explicit goal of strengthening government capacity. To many respondents, contributing to state building has meant taking care not to undermine the authority of state actors while ensuring humanitarian support is delivered in a neutral manner.

#### Processes

Respondents generally agreed about the criticality of decision-making being inclusive, however there were challenges in striking the right balance of inclusivity. In particular, decisions around flight restrictions and lockdown measures were said to be done without broad consultation and participation appears to have been limited to some federal cabinet members.

“*We were informed probably a day and a half or a day I would say if my memory serves me right*, *that there would be on lockdown and to make the necessary arrangements”–*UN Official 6.

Other respondents indicated short-cutting typical inclusive approaches in favour of speeding up the decision-making process. Some respondents, primarily donors, mentioned simplifying their decision-making process by giving more leeway to implementing partners to conduct activities without the typical numerous rounds of consultation.

*“We didn’t have the time to go through the wholly inclusive approach*, *as we would for the HRP*, *but we did engage with the ministry*, *with the lead agencies*, *and several of the larger more active partners to see what they were thinking about some of these things”* UN Official 2

In navigating the complexities of the response, many actors discussed their reliance on expert advice. Most respondents mentioned utilizing formal channels to seek expert advice internally or particularly from the World Health Organization (WHO). Some respondents suggested that some of the expert advice provided, particularly around a plethora of mathematical models and projections was overwhelming and not well understood.

*I remember in those early days*, *there was a lot of time spent analysing these things*. *Maybe [we were] not equipped really to use them effectively*.–Donor Official 4

Respondents mentioned struggling to determine where COVID-19 stood in relation to threats from competing emergencies. Some respondents affirmed utilizing a no-regrets approach in prioritizing COVID-19 which in practical terms manifested in COVID-19 trumping all other health priorities in the country.

*Of course when COVID began not only in Somalia but everywhere*, *the focus was on how to contain this virus and how to treat people*. *It is true that there was a shift taken from all other areas and all efforts and resources directed to COVID*, *attention was diverted away from areas like maternal and child vaccination services in Somalia*, *TB*, *malaria and HIV-* UN Official 3

A number of respondents however indicated utilizing a more pragmatic and cautious approach through attempting to maintain a minimum of essential services throughout the epidemic. Additionally, some respondents stated favouring a preventative strategy because they did not have the capacity to conduct large scale case management.

*“I think we try to do the minimum basics which are protect essential services*, *equip health workers*, *do infection prevention within the basics and recognize that maybe some of these secondary impacts get more significant and then watch and see what happened*. *Yes*, *we definitely avoided the swing of the whole program*. *“-*Donor Official 4

Respondents described activating existing organizational mechanisms as part of the analysis process.

*“Whenever a crisis situation happens*, *we normally activate our Country Emergency Teams*, *led by the country emergency coordinator*. *They do the quick assessments and that kind of stuff*, *and that information is immediately disseminated to the regional and HQ level*, *for the information*, *with all the analysis”–*NGO Official 4

Respondents mentioned being guided by these mechanisms and protocols to quickly analyse the severity of the situation, the decisions to be taken and whether local capacity is sufficient to deal with the crisis or whether external supported is warranted. A limited number of respondents stated that their organisation’s use of contingency plans and funds in the acute phase of the epidemic allowed them to make efficient and timely decisions.

### Response: Arriving at decisions

After making sense of the situation and conducting their analyses, respondents described arriving at a number of decisions to adopt overall approaches as well as to undertake specific activities.

Responders outlined a number of limitations that they felt impacted on the timeliness and performance of response measures in Somalia. These included lack of capacity, resource limitations, physical access to populations, political considerations, as well as difficulties engaging with the public.

Lack of capacity of state authorities to implement COVID-19 measures was the most commonly cited limitation by responders. This included capacity to roll out wide-scale testing and influence and enforce certain population measures such as the wearing of face masks, the closure of mosques and curfews.

Respondents emphasized the importance of flexibility and adaptability in harnessing the high-level attention directed at COVID-19 to respond effectively without undermining existing humanitarian response. In particular, they mentioned adapting ongoing programmes to ensure continuity of lifesaving activities.

“*We did not overwhelmingly put the COVID there as enemy number one of Somalia*. *That is not the case*. *We pleaded*, *and I think many of the other donors pleaded for continuation of the normal programs as much as possible*, *with here and there an adaptation and here and there additional funding for the COVID*, *but the most essential thing was to keep going with the other programs*.*”–*Donor Official 1

Despite a majority of donor funding being earmarked for specific humanitarian or development projects, donor flexibility in shifting to COVID-19 response was also highlighted by respondents. Respondents mentioned utilizing crisis modifier mechanisms (legal clauses permitting program adjustments) to redirect resources. However, despite these mechanisms, this process was not as fast as required nor was it fit for pandemic response.

Respondents also mentioned putting in place organizational business continuity measures. Measures included adopting remote working arrangements, designating replacements for incapacitated staff members, shielding vulnerable staff, as well as producing and implementing business continuity plans.

Importantly, respondents stated locally contextualizing and adapting global response measures to circumstances within Somalia. Some respondents mentioned three stages of localization: adapting guidance from headquarters to regional contexts, from regional to Somalia context and within Somalia, adapting from state to state.

Respondents also described adapting and refining COVID specific response measures to improve effectiveness, for example in the targeting of relevant age groups. Further instances of adaptation included modifying community surveillance systems to include COVID-19, modifying programme delivery to maintain social distance, using locally available resources to produce masks and PPE as well as test for COVID-19 using GeneXpert machines already available in the country for tuberculosis control.

In previous localized epidemics, respondents stated utilizing a bottom-up approach towards the decision-making and response.

“*If I recall the 2016*, *2017 cholera outbreak*, *the decision rather came from this level*, *the country level*, *up the ladder to ask for support*. *Because it was localized cholera in Somaliland and Puntland”–*Red Cross Official 1.

In contrast, some respondents suggested that early in the epidemic, response priorities and activities were largely set at a global headquarter or regional level but as time progressed, the local counterparts took over this role.

## Discussion

From the perspective of decision makers, the catastrophic scenario of the epidemic seemed not to have materialized in Somalia. However, this may be less to do with response efforts and more to do with population characteristics (e.g. age, outdoor social mixing) [39] and/or inadequate surveillance. Anecdotal reports as well as recent studies suggest that the impact in terms of mortality was indeed much higher than reported [40,41]. As such it is even more crucial to reflect on the decisions that impacted on this outcome. The largely acritical adoption of response measures developed abroad in the early period of the epidemic may be characterised as emerging from conformity bias during the ‘sense-making’ phase of decision-making. This type of bias in which decision makers in Somalia emphasized conforming to international response norms has been observed in other humanitarian crises [21]. This conformity occurring despite Somalia’s ample experience with outbreaks and well-established humanitarian mechanisms illustrates the exceptional nature of the COVID-19 pandemic. Excessive effort and resources were directed at case management relative to Non-Pharmaceutical Interventions such as risk communication & community engagement [28]. No serious consideration of alternatives to lockdown and blanket population restrictions such as shielding of vulnerable people [42] took place at the outset due to a number of factors. These include the perception that it would be complicated to communicate such alternatives and would likely result in low adherence. The centralized approach adopted by the federal government was also an important characteristic of the response though it was not unique to Somalia and seems to have been the preferred method by many governments [43]. However, in the case of Somalia, the overcentralization of the response risked eroding the already tenuous capacity of the various line ministries to conduct their work.

Discord among various actors often constrained the ‘analysis’ phase of decision-making for the COVID-19 response in Somalia. The epidemic occurring in a heated election year and political manoeuvring and contestation was an important feature of response dynamics [44]. Such politicized decision-making environments have been known to lead to polarisation [45] and may hinder response cooperation. The occurrence of election clashes despite pandemic restrictions presents an example of such polarisation [46].

In terms of outcomes, decisions early in the response may have contributed to the direct and indirect impact of the epidemic disproportionally being felt by the poorest segments of society [47]. Authorities attempted to offset this impact by instituting price controls on food, tax reductions alongside the maintenance and expansion of cash programmes by some actors [48]. The fall in utilization of vaccination and maternal health services illustrates that decision-makers may have been unsuccessful in balancing these key priorities against COVID-19 response [49]. The decision to loosen restrictions at the end of the first wave including permitting diaspora arrivals from high-burden countries may also have contributed to resurgence of the virus and accelerated introduction of new variants [50].

New pandemic waves are likely due to low vaccination coverage and the emergence of new variants that may partially evade immunity. As a result, there is an urgent need to improve the decision-making process in light of this threat. Currently, the decision-making around COVAX has been is limited to a few agencies with few individuals involved in the planning. Inclusive and open decision-making can contribute to strengthening public trust and legitimacy [51] and is key to addressing the limited uptake of the vaccines in Somalia [52]. The decision to administer vaccines within health facilities ignores considerable vaccine hesitancy among the population and sets aside the substantial experience of health actors in delivering community-based vaccine drives. Mobile and outreach approaches should be utilized as has been recommended in crisis-effected settings [53].

### Appropriateness of decision-making framework

**Fig 3:**
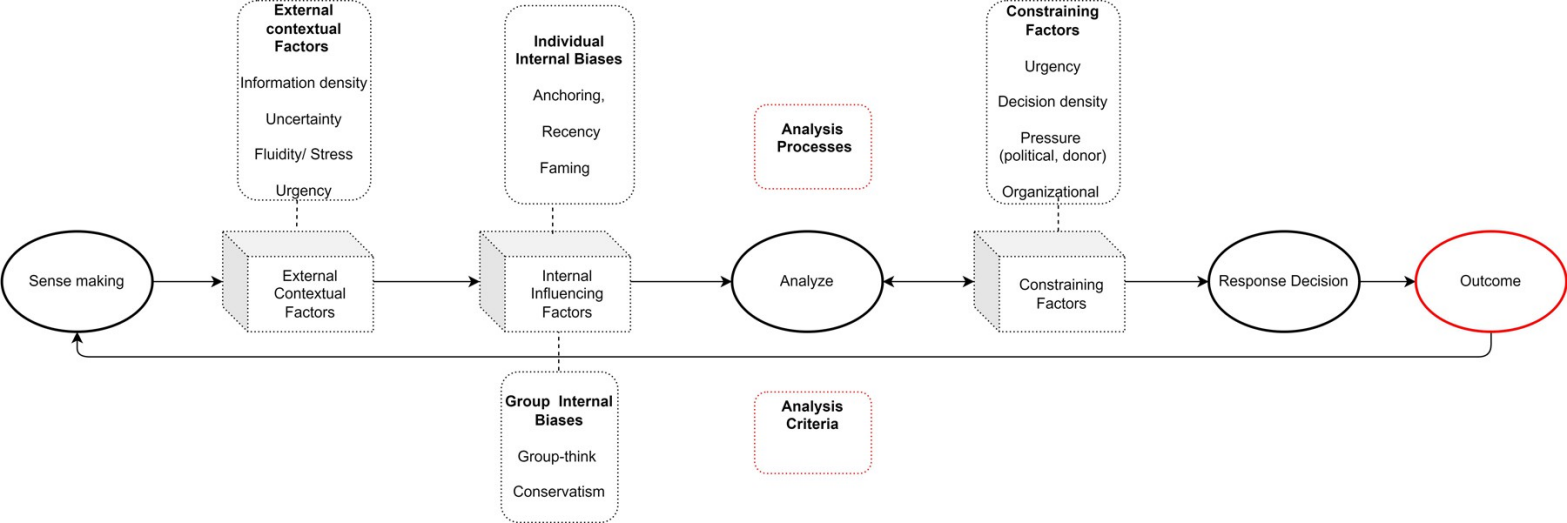
Updated decision-making framework for epidemic response.

The DMF was largely found to be valuable in conceptualizing the decision-making arena in the Somali COVID-19 response. This study allowed for additional dimensions to be added to the framework including further refinement of the analysis element into processes, criteria and constraints (Fig 3). Additionally, the results indicated a need to make a clear distinction between response decisions and their outcomes.

## Recommendations

This study revealed the outsized influence that the decision-making process had on the shape of the COVID-19 response in Somalia. It is therefore important in an epidemic context to strengthen the process by which decision makers arrive at their response decisions. This process includes embedding best deliberative practice in order to account for individual and group preferences or biases. Such best practice includes increasing transparency around the evidence utilized and the decision-making processes implemented as well as clarifying roles and responsibilities. Furthermore, it is critically important to devolve decision-making to allow for robust technical discussion and consensus building. One way this can be done is to ensure a more diverse range of stakeholders as well as to allow for divergent opinions to be thoroughly debated. Strengthened decision-making processes should allow for participation by skill and experience rather than by rank. Decision makers should also be more explicit about uncertainties and motivations in order to more openly and effectively address them. Lastly decision-makers would benefit from decision support tools which incorporate these recommendations and would allow for more effective and timely epidemic response.

## Conclusion

The challenges and opportunities of the covid-19 response in Somalia typifies what other low-income and crisis-affected countries may potentially be facing. Such settings not only must deal with the epidemic directly and its indirect impacts but must also contend with a host of urgent competing emergencies. As such decision-makers are under heavier strain than those in better resourced and stable environments and require adequate support. COVID-19 is unlikely to be the last pandemic faced by decision-makers in such settings and therefore evaluations such as this are especially critical to strengthening future preparedness and improving response.

## Supporting information

S1 Search Strategy Search Strategy underlying the grey literature search. Table A grey literature English search terms. Table B grey literature Somali search terms.
(DOCX)
