# Supplementary material for: Evaluating COVID-19 decision-making in a humanitarian setting: The case study of Somalia
Source: PLOS Glob Public Health. 2022 Mar 16;2(3):e0000192. doi: 10.1371/journal.pgph.0000192 (PMC10021687; doi:10.1371/journal.pgph.0000192)
Supplement: S1 Search Strategy — Table A grey literature English search terms. Table B grey literature Somali search terms. (DOCX) [file pgph.0000192.s001.docx]

# Supplementary Material

## Grey Literature Search Strategy

Search Engines: Google.com, scholar.google.com

Websites: reliefweb.int, humanitarianresponse.info , WHO Somalia, Ministry of Health Somalia, WHO EMRO, alnap.org,

Timeframe: 2020-2021

Search terms

Language: English

Table A Grey literature English search terms

| **Geographic** | **Disease** | **Decision** | **Documents** |
| --- | --- | --- | --- |
| Somalia | COVID-19 | Decisions | evaluation |
| Mogadishu | COVID | Options | Response plan |
| Puntland | Coronavirus | Decision-making | Sitrep/ Situation report |
| Somaliland | pandemic | Decision making | Bulletin |
|  |  |  | assessment |

Search queries

1. Somalia OR Mogadishu OR Puntland OR Somaliland
2. COVID-19 OR COVID19 OR COVID OR Coronavirus
3. Decision* OR Option OR Decision-making OR decision making
4. Evaluation or Response plan OR Sitrep OR Situation report OR Bulletin or Assessment

Combined: 1 & 2 & 3 & 4

Language: Somali

Table B Grey literature Somali search terms

| **Geographic** | **Disease** | **Decision** | **Documents** |
| --- | --- | --- | --- |
| Somalia | COVID-19 | Go’aan | qiimaynta |
| Mogadishu | COVID | Go’aama | Warbixinta xaalada |
| Puntland | Fayruuska korona | go'aan qaadashada |  |
| Somaliland |  |  |  |

1. Somalia OR Mogadishu OR Puntland OR Somaliland
2. COVID-19 OR COVID19 OR Fayruska Korona OR Coronavirus
3. Go’aan* OR Go’aama OR Go’aan qaadashada
4. Qiimaynta OR Warbixinta xaalda

Combined: 1 & 2 & 3 & 4
